# Deep Learning-Based Screening Test for Cognitive Impairment Using Basic Blood Test Data for Health Examination

**DOI:** 10.3389/fneur.2020.588140

**Published:** 2020-12-14

**Authors:** Kaoru Sakatani, Katsunori Oyama, Lizhen Hu

**Affiliations:** ^1^Department of Human and Engineered Environmental Studies, Graduate School of Frontier Sciences, The University of Tokyo, Tokyo, Japan; ^2^Institute for Healthcare Robotics, Future Robotic Organization, Waseda University, Tokyo, Japan; ^3^Department of Computer Science, College of Engineering, Nihon University, Tokyo, Japan

**Keywords:** Alzheimer's disease, artificial intelligence, deep leaning, dementia, Mini Mental State Examination, screening test, vascular cognitive impairment

## Abstract

**Background:** In order to develop a new screening test of cognitive impairment, we studied whether cognitive function can be estimated from basic blood test data by applying deep learning models. This model was constructed based on the effects of systemic metabolic disorders on cognitive function.

**Methods:** We employed a deep neural network (DNN) to predict cognitive function based on subject's age and blood test items (23 items). We included 202 patients (73.48 ± 13.1 years) with various systemic metabolic disorders for training of the DNN model, and the following groups for validation of the model: (1) Patient group, 65 patients (73.6 ± 11.0 years) who were hospitalized for rehabilitation after stroke; (2) Healthy group, 37 subjects (62.0 ± 8.6 years); (3) Health examination group, 165 subjects (54.0 ± 8.6 years) admitted for a health examination. The subjects underwent the Mini-Mental State Examination (MMSE).

**Results:** There were significant positive correlations between the predicted MMSE scores and ground truth scores in the Patient and Healthy groups (*r* = 0.66, *p* < 0.001). There were no significant differences between the predicted MMSE scores and ground truth scores in the Patient group (*p* > 0.05); however, in the Healthy group, the predicted MMSE scores were slightly, but significantly, lower than the ground truth scores (*p* < 0.05). In the Health examination group, the DNN model classified 94 subjects as normal (MMSE = 27–30), 67 subjects as having mild cognitive impairment (24–26), and four subjects as having dementia (≤ 23). In 37 subjects in the Health examination group, the predicted MMSE scores were slightly lower than the ground truth MMSE (*p* < 0.05). In contrast, in the subjects with neurological disorders, such as subarachnoid hemorrhage, the ground truth MMSE scores were lower than the predicted scores.

**Conclusions:** The DNN model could predict cognitive function accurately. The predicted MMSE scores were significantly lower than the ground truth scores in the Healthy and Health examination groups, while there was no significant difference in the Patient group. We suggest that the difference between the predicted and ground truth MMSE scores was caused by changes in atherosclerosis with aging, and that applying the DNN model to younger subjects may predict future cognitive impairment after the onset of atherosclerosis.

## Introduction

As the world's population ages rapidly, dementia is becoming a major global health problem. Currently, emphasis is being placed on early diagnosis and intervention to prevent dementia onset (1). Therefore, a screening test for cognitive dysfunction is important for early diagnosis. At present, the Mini Mental State Examination (MMSE) is the most commonly used test for cognitive function evaluation (2, 3). The MMSE is an accurate and cost-effective screening test. However, it is impractical to screen a large number of subjects over a short time span. The reasons being that it is subjective in nature and conducted on a one-to-one basis between the inspector and subject. Moreover, it is difficult administering the test to subjects with disorders such as visual and hearing impairments.

Peripheral biomarkers such as amyloid β are used as biomarkers of Alzheimer's disease (AD) (4). However, these biomarkers originate from cerebrospinal fluid and are not suitable for mass screening because they require an invasive lumbar puncture. Although a number of studies investigating AD-related biomarkers in blood samples have been conducted, the results are still inconclusive (5). In addition, amyloid alone is not sufficient to account for the dementia syndrome; clearance of amyloid plaques in AD by immunization with Aβ42 did not prevent progressive neurodegeneration (6). Consequently, there is a growing demand for alternative peripheral biomarkers in a more readily accessible sample material for screening tests.

In order to develop a new screening test for dementia, we focused on the relationship between systemic disorders and cognitive impairment. For example, vascular cognitive impairment (VCI), which is caused by arteriosclerosis based on lifestyle-related diseases, plays an important role in cognitive disorders ranging from mild cognitive impairment (MCI) to severe dementia (7–9). Importantly, vascular pathology is believed to contribute not only to vascular dementia, but also to AD; magnetic resonance imaging (MRI) has demonstrated that small-vessel cerebrovascular disease (i.e., white matter hyperintensities) caused by atherosclerosis contributes to the presentation of AD and is necessary for the its clinical manifestation (10). Other systemic disorders that could affect cognitive function are metabolic disorders, including malnutrition (11), anemia (12), lipid metabolism (13), purine metabolism (14), and renal function impairment (15). Importantly, these systemic disorders can be detected via basic blood tests of health examination.

In the present study, in order to develop a new screening test of cognitive impairment, we studied whether cognitive function can be estimated from basic blood test data by applying deep learning models. This model was constructed based on the effects of systemic metabolic disorders on cognitive function. To analyze the complex non-linear relationships between blood test data and cognitive function, we used a deep neural network (DNN), which allows the analysis of regularity and relevance from a large amount of data to make judgments and predictions (16). In the field of life sciences, DNNs have been applied to various medical fields, such as imaging diagnosis (17–19). In the field of dementia, DL was applied to the computer-aided diagnosis of AD based on MRI images (20). However, MRI is not suitable for mass dementia screening. In the next step, we validated the prediction accuracy of the DNN model using a leave-one-out cross-validation (LOOCV) in the training group. Then, we validated the accuracy in subjects that were not used for training the DNN model. Finally, we evaluated the clinical usefulness of the DNN model as a screening test of dementia by applying the algorithm.

## Subjects and Methods

### Subjects

We included a total of 202 patients (87 men, 115 women; mean age ± SD, 73.48 ± 13.1 years) who were admitted to the Southern Tohoku Kasuga Rehabilitation Hospital (Sukagawa City, Japan) for training the DNN model of cognitive function; all subjects received various hospitalization treatments, including rehabilitation and medication of lifestyle diseases. Table 1 shows the clinical profiles of the patients: 142 patients (68.8%) had cerebrovascular diseases (cerebral infarction, 79 cases; cerebral hemorrhage, 41 cases; subarachnoid hemorrhage, 21), while 174 patients (94.6%) had at least one life-style disease.

**Table 1:** Clinical profiles of the patients.

	**Types of lifestyle diseases**													**Total**
	**HT**	**DM**	**HL**	**HT, DM**	**DM, HL**	**HT, HL**	**HT, G**	**HL, G**	**HT, HL DM**	**HT, HL DM, G**	**HT, DMG**	**HT, HL, G**	**None**	
CH	18 (7)	1 (0)	0 (0)	6 (5)	0 (1)	6 (1)	0 (1)	0 (0)	6 (0)	1 (0)	0 (0)	0 (0)	3 (1)	41 (16)
CI	16 (7)	3 (2)	6 (2)	12 (2)	0 (4)	10 (3)	2 (0)	2 (1)	14 (1)	1 (0)	1 (1)	5 (1)	7 (5)	79 (29)
SAH	9 (2)	0 (0)	4 (0)	1 (0)	0 (0)	3 (2)	0 (0)	0 (0)	3 (0)	0 (0)	0 (0)	0 (0)	1 (2)	21 (6)
HI	2 (0)	0 (0)	0 (0)	0 (0)	0 (0)	0 (0)	0 (0)	0 (0)	0 (0)	0 (0)	0 (0)	0 (0)	1 (0)	3 (0)
BF	16 (2)	1 (0)	0 (0)	4 (0)	0 (0)	4 (1)	0 (0)	0 (0)	2 (0)	0 (0)	0 (0)	0 (0)	12 (2)	39 (5)
Others	7 (1)	0 (0)	0 (1)	4 (0)	0 (0)	0 (2)	0 (0)	0 (0)	1 (1)	0 (0)	1 (0)	1 (0)	5 (4)	19 (9)
Total	69 (19)	5 (2)	10 (3)	27 (7)	0 (5)	23 (9)	2 (1)	2 (1)	26 (2)	2 (0)	2 (1)	6 (1)	31 (14)	202 (65)

*HT, hypertension; DM, diabetes mellitus; HL, hyperlipidemia; G, gout; CH, cerebral hemorrhage; SAH, subarachnoid hemorrhage; CI, cerebral infarction; HI, head injury; BF, born fracture*.

*Numbers indicate the number of patients for the training of DNN model while numbers in parentheses patients indicate for validation of the accuracy of the DNN model*.

In order to validate the accuracy of the DNN model, we studied a total of 267 subjects who were not used for training the DNN model. The subjects included the following three groups: (1) Patient group, 65 patients (32 men, 33 women; 73.6 ± 11.0 years) who were hospitalized for rehabilitation after stroke in Southern Tohoku Kasuga Rehabilitation Hospital (Sukagawa City, Japan) (Table 1). (2) Healthy group: 37 healthy subjects (six men, 31 women; 62.0 ± 8.6 years) who were members of a sports gym (Sakura) attached to Southern Tohoku Kasuga Rehabilitation Hospital (Sukagawa City, Japan). (3) Health examination group: 165 subjects (83 men, 82 women, 54.0 ± 8.6 years) who visited the outpatient clinic of dementia prevention (K.S. the doctor in charge) at the Tokyo Clinic (Tokyo, Tokyo).

The present study was approved by the Life Science Research Ethics and Safety of the University of Tokyo (Approval Number: 19-318).

### Blood Test

We performed a blood test in all the subjects, including a complete blood count and basic metabolic panel, at the time of the experiment. The blood test items are shown in Table 2. The blood test data were put into the input layer of the DNN model.

**Table 2:** Blood test items for the prediction of cognitive function.

**Complete blood count**	**General biochemical examination**	
WBC count	Total protein	BUN
RBC count	Albumin	Creatinine
Hemoglobin	A/G ratio	Uric Acid
Hematocrit	AST (GOT)	Glucose
MCV	ALT (GPT)	Na
MCH	r-GTP	K
MCHC	Total Cholesterol	CI
Platelet count	Triglyceride	

*MCV, mean corpuscular volume; MCH, mean corpuscular hemoglobin; MCHC, mean corpuscular hemoglobin concentration; BUN, blood urea nitrogen*.

### Assessment of Cognitive Function

We assessed the cognitive dysfunction of the subjects using the Mini Mental State Examination-Japanese (MMSE-J) (21). The MMSE scores were put into the output layer of the DNN model as the ground truth data. In order to select patients with suspected cognitive impairment or dementia, we used a cut-off value of 23/24 (22). By applying the cut-off value to the DNN model's output, we derived a binary classification model to predict the presence or absence of cognitive impairment.

### Magnetic Resonance Imaging

In order to evaluate the relationship between the blood test data and changes in anatomical structure, 40 subjects (23 subjects in the Inpatient group and 17 subjects in the Health examination group) underwent a magnetic resonance imaging (MRI) scan using a 1.5-T Vision Plus imager (Siemens, Erlangen, Germany). One hundred and forty 3D sections of a T1-weighted magnetization-prepared rapid acquisition of gradient echo sequence were obtained in the sagittal orientation with 1.2-mm thick sections (field of view = 23, repetition time = 9.7 ms, echo time = 4 ms, flip angle = 12°, and inversion time = 300 ms, with no intersection gaps).

We analyzed morphological changes in the brain using a voxel-based specific regional analysis system for Alzheimer's disease (VSRAD), a diagnosis-aiding program that runs on Windows, for voxel-based morphometry based on statistical parametric mapping (SPM8) and diffeomorphic anatomical registration using the exponentiated lie (DARTEL) (23). VSRAD is widely used in current clinical practice for the treatment of AD (24).

VSRAD generates the following scores (23): (1) Severity, the severity of atrophy obtained from the averaged positive z-score in the target volume of interest (VOI) (i.e., hippocampus and its surroundings); (2) Extent (%), the extent of a region showing significant atrophy in the target VOI—i.e., the percentage of coordinates with a *z*-value exceeding the threshold value of 2 in the target VOI; (3) Ratio, the extent of a region showing significant atrophy in the whole brain—i.e., the percentage of coordinates with a *z*-value exceeding the threshold value of 2 in the whole brain; (4) Whole Brain Extent (%), the ratio of the extent of a region showing significant atrophy in the target VOI to the extent of a region showing significant atrophy in the whole brain.

### Data Analysis

First, we measured the Pearson correlations between MMSE scores and individual blood test items in the training set. We likewise measured the correlation between subject age and MMSE score.

Second, we employed multivariate regression analysis to evaluate the factors (i.e., age, sex, and blood test items) that correlated with cognitive function. In addition, we assessed the risk factors of cognitive impairment using logistic regression analysis. Finally, based on the two-class classification of normal subjects (MMSE ≥24) and subjects with cognitive impairment (MMSE ≤23), we evaluated the accuracy of the MMSE scores predicted by logistic regression analysis.

**Figure 1:**
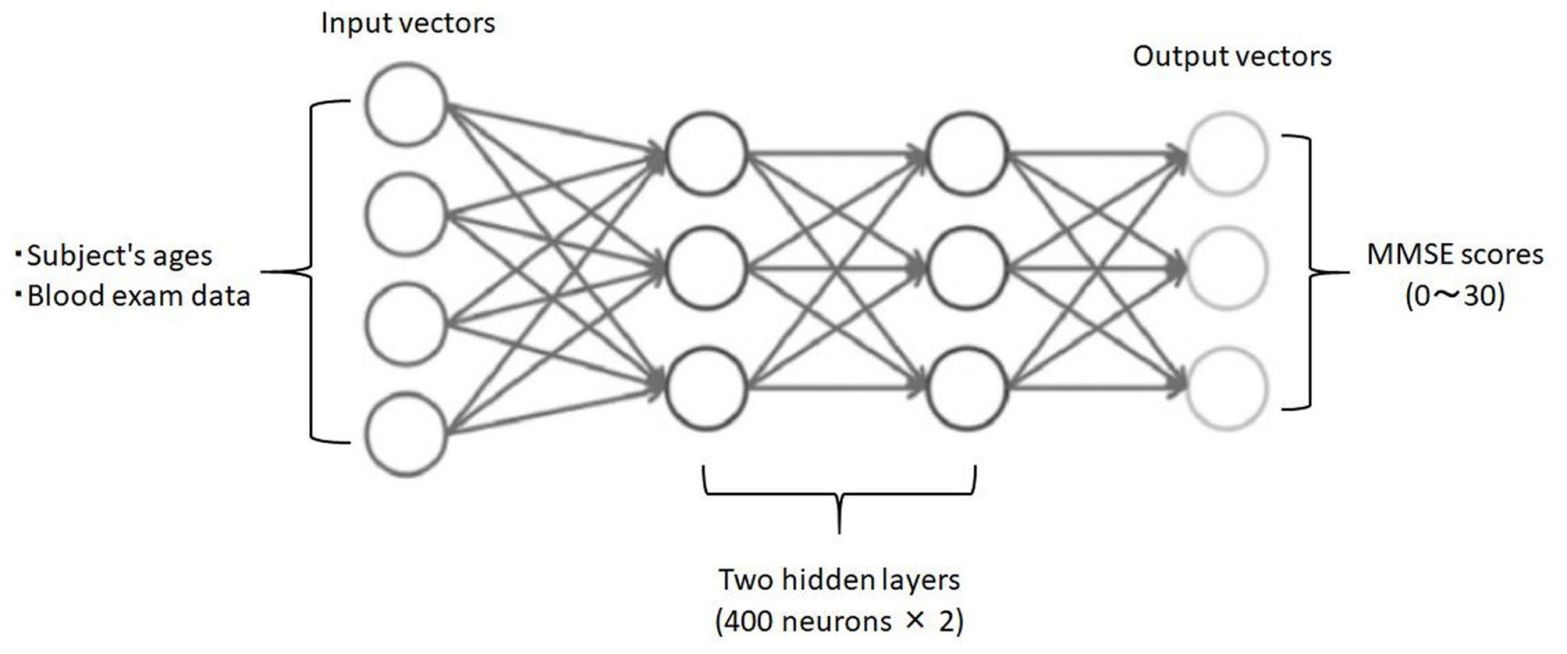
Schematic drawing of the DNN model for predicting MMSE scores. Input vectors include subject age and blood examination data. The output vector is a regression to estimate the MMSE score. The hidden layer contains no backward connections from the downstream layers.

Third, employing deep neural networks (DNNs) as deep learning algorithm, we analyzed the relationship between blood data (input) and the ground truth MMSE scores in the subjects (output). To implement the DNN, we used the H2O open source machine learning library (25, 26). H2O allows the configuration of multilayer feedforward neural networks. Input to the DNN in this study consisted of 25 variables, including subject age plus 23 blood test items in the input layer (Figure 1). The DNN has two hidden layers with 400 neurons each. The weighted combination $\alpha =\overset{n}{\underset{i=1}{\sum }}w_{i}x_{i}+b$ aggregates the input signals *x*_*i*_ in each layer to activate an output signal *f*(α) to the connected neuron in the next layer. The function *f* represents the non-linear activation function used throughout the network, and the bias *b* accounts for the neuron's activation threshold. The output signal *f*(α) in each layer is thus determined by the weighted combination with the input signals *x*_*i*_ from the higher layers of the DNN. In the output layer, a loss function *L*(*W, B* | *j*) is measured using the mean square error between the estimated value and the actual MMSE score. The learning process updates the weights *W* and biases *B* until the loss function *L*(*W, B* | *j*) is minimized. We use *W* to denote the collection{_*W*_*i*_}1:*N*−1_, where *W*_*i*_ denotes the weight matrix connecting layers *i* and *i*+1 for a network of *N* layers. Similarly, *B* denotes the collection{_*b*_*i*_}1:*N*−1_, where *b*_*i*_ denotes the column vector of biases for layer *i*+1. The algorithm was validated by LOOCV.

Finally, the DNN and the logistic regression analysis were compared for predictive accuracy of the two-class classification of MMSE scores (i.e., the cut-off value of 23/24).

## Results

### Pearson Correlations Between MMSE Scores and Blood Test Data

**Figure 2:**
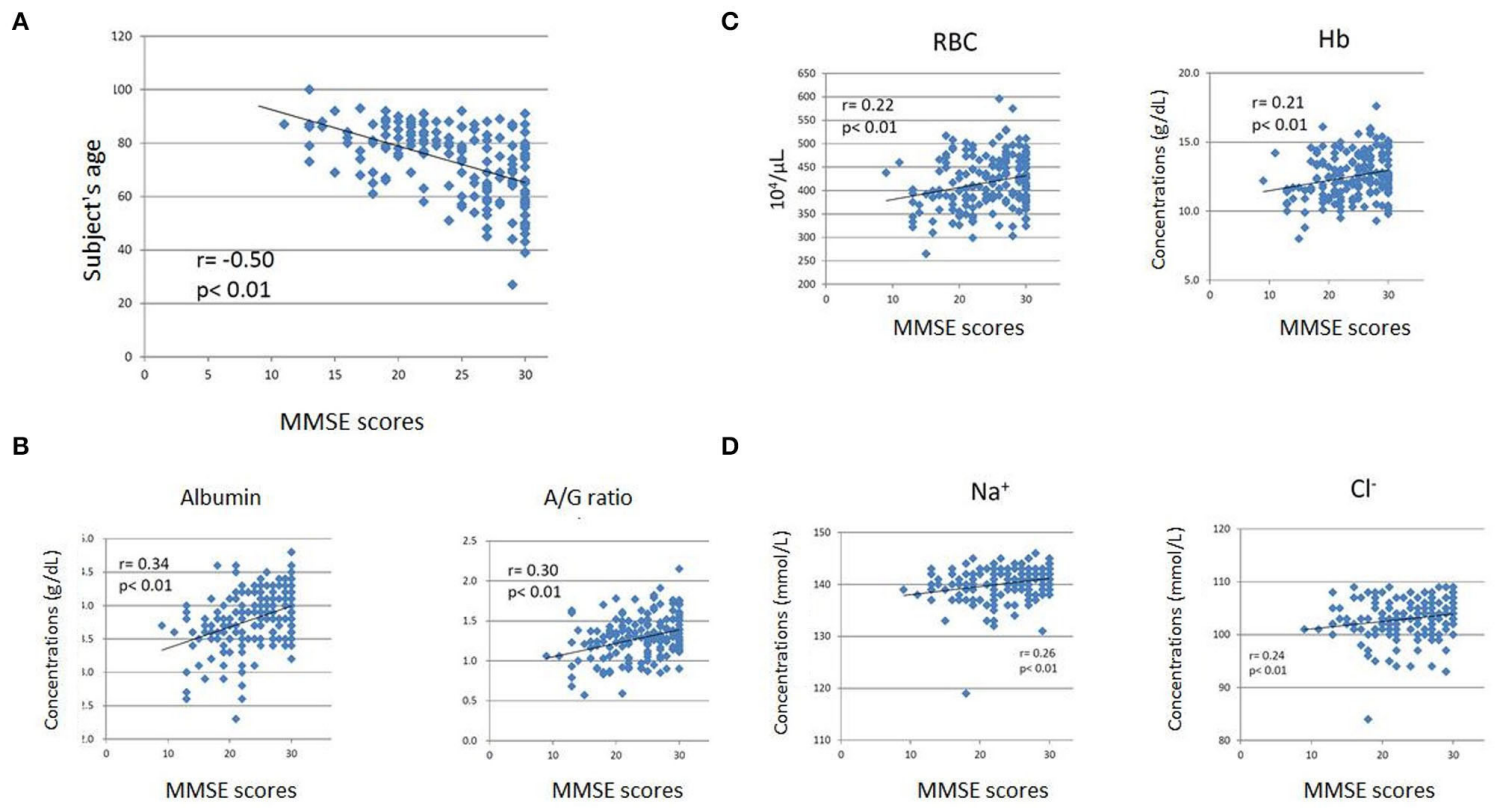
Scatter plots of Mini-Mental State Examination (MMSE) scores and subjects' ages **(A)**, albumin level and A/G ratio **(B)**, RBC count and Hb level **(C)**, and sodium (Na) and chloride (Cl) levels **(D)**. The subjects' age exhibited a significant negative correlation with MMSE scores **(A)**. The blood test items exhibited weak but significant positive correlations with MMSE scores **(B–D)**. The horizontal axes indicate MMSE scores, while the vertical axes indicate age **(A)** and concentrations (B-albumin, **C,D**).

We observed the following correlations between subject age, blood test data, and cognitive function measured by MMSE. Subject age exhibited a significant negative correlation with MMSE scores (*r* = −0.50, *p* < 0.01) (Figure 2A). Albumin (*r* = 0.35, *p* < 0.01) and the A/G ratio exhibited significant positive correlations with MMSE scores (Figure 2B). In addition, red blood cell count (RBC) (*r* = 0.23, *p* < 0.05), hemoglobin concentrations (Hb) (*r* = 0.22, *p* < 0.05), and hematocrit (Ht) (*r* = 0.24, *p* < 0.05) were significantly positively correlated with MMSE scores (Figure 2C). Furthermore, sodium (Na) (*r* = 0.32, *p* < 0.01) and chloride (Cl) (*r* = 0.22, *p* < 0.05) showed significant positive correlations with MMSE scores (Figure 2D). Finally, triglyceride (TG) (*r* = 0.24, *p* < 0.05) and uric acid (UA) (*r* = 0.22, *p* < 0.05) were significantly positively correlated with MMSE scores.

### Multivariate Regression Analysis and Logistic Regression Analysis

Employing multivariate regression analysis, we evaluated the factors (i.e., age, sex, and blood test items) that correlated with cognitive function (Table 3). We found that cognitive function expressed by the MMSE score decreased with increasing age; the partial regression coefficient was −0.17 (*p* < 0.001). In addition, cognitive function increased as PLT, total protein, and CI increased; partial regression coefficients were 0.179 (*p* < 0.01), 1.803 (*p* < 0.05), and 0.523 (*p* < 0.001), respectively.

**Table 3:** Multiple regression analysis of variables with regard to cognitive function.

**Variable**	**Coefficient**	**Confidence interval**		**Std. error**	***P*-value**	
		**2.5%**	**97.5%**			
Age	−0.170	−0.232	−0.109	0.031	0.000	^***^
WBC	0.000	−0.001	0.000	0.000	0.140	
RBC	0.013	−0.001	0.028	0.007	0.076	
PLT	0.179	0.061	0.298	0.060	0.003	^**^
Total protein	1.803	0.419	3.188	0.702	0.011	^*^
CI	0.528	0.309	0.747	0.111	0.000	^***^

* *p < 0.05*,

** *p < 0.01*,

*** *p < 0.001*.

Table 4 shows the risk factors of cognitive impairment evaluated by logistic regression analysis. The risk of cognitive impairment increased with age, with an odds ratio of 1.104 (*p* < 0.001). The risk of cognitive impairment decreased with increases in PLT, total protein, and CI; the odds ratios were 0.91 (*p* < 0.01), 0.453 (*p* < 0.05), and 0.783 (*p* < 0.001), respectively.

**Table 4:** Risk factors of cognitive impairment evaluated by logistic regression analysis.

**Variable**	**Coefficient**	**Std. error**	**Odds ratio**	**Confidence interval**		***P*-value**	
				**2.5%**	**97.5%**		
Age	0.099	0.020	1.104	1.061	1.149	0.000	^***^
PLT	−0.094	0.031	0.910	0.857	0.967	0.002	^**^
Total protein	−0.793	0.392	0.453	0.210	0.977	0.043	^*^
Albumin	−0.888	0.539	0.412	0.143	1.182	0.099	
K	−0.517	0.386	0.596	0.280	1.271	0.180	
CI	−0.245	0.068	0.783	0.685	0.894	0.000	^***^
Glucose	0.014	0.010	1.014	0.994	1.035	0.171	

* *p < 0.05*,

** *p < 0.01*,

*** *p < 0.001*.

Based on the two-class classification of normal subjects (MMSE ≥24) and subjects with cognitive impairment (MMSE ≦ 23), we evaluated the accuracy of the MMSE scores predicted by logistic regression analysis. We found that the prediction accuracy for cognitive impairment (MMSE ≤ 23) was 74.16%, for normal cognitive function (MMSE ≧ 24) was 78.64%, and the estimation rate was approximately 76.56%.

### Prediction of MMSE Scores Using the DNN Model

**Figure 3:**
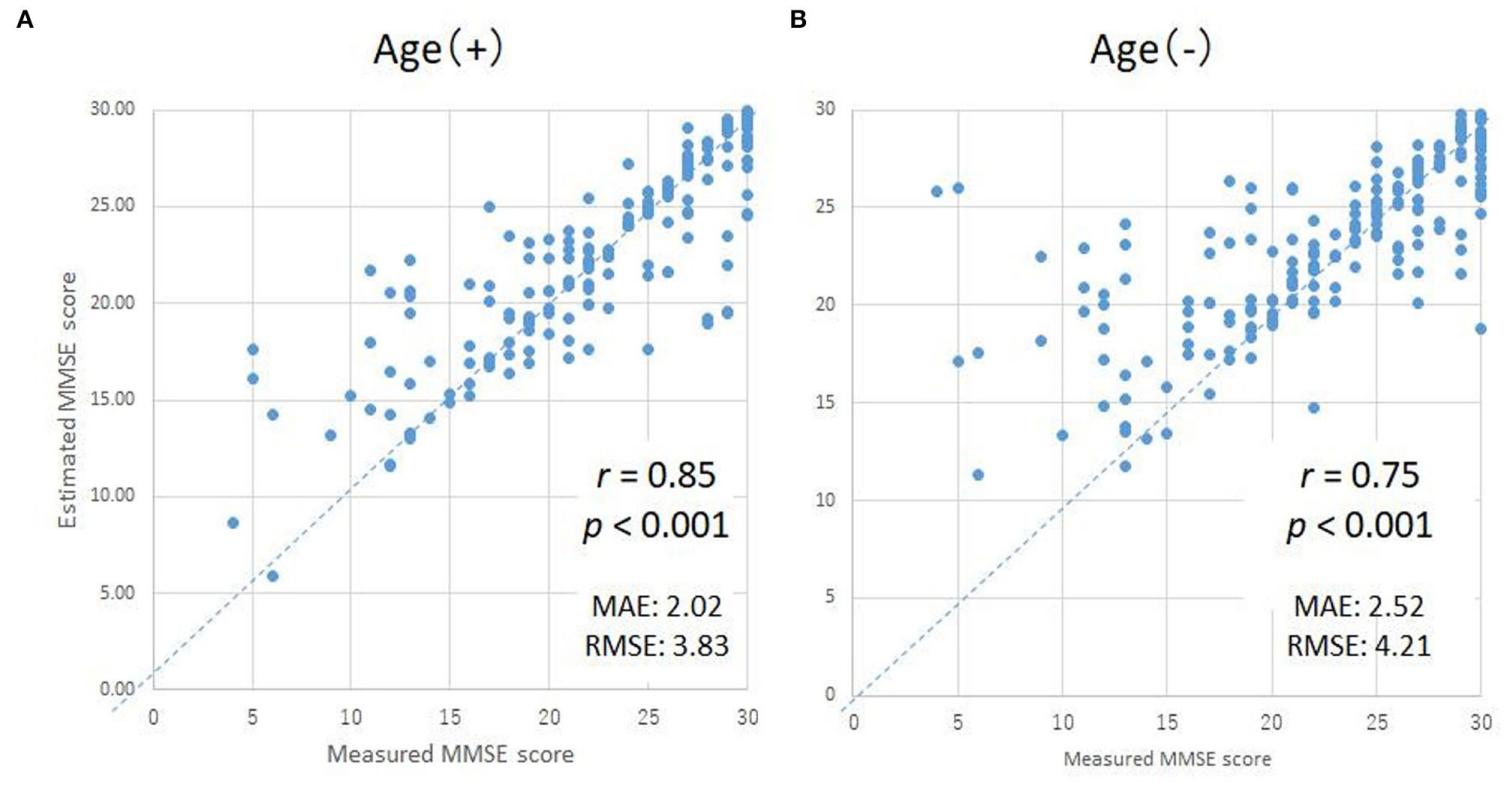
Scatter plots of the measured and predicted MMSE scores by DNN with age **(A)** and without age **(B)**. The horizontal axes indicate the measured MMSE scores, while the vertical axes indicate the predicted MMSE scores by DNN.

In order to validate the DNN model, we compared the ground truth MMSE scores with those predicted by the DNN model using LOOCV in the training group. The predicted MMSE scores exhibited a significant positive correlation with the ground truth MMSE scores (*r* = 0.85, *p* < 0.001) (Figure 3A). The mean absolute error was 2.02, while the root mean square error was 2.02, as compared to the raw scores, which ranged from zero to 30.

### Effects of Aging on Predicted MMSE Scores Using the DNN Model

Since subject age exhibited the highest correlation coefficient, the MMSE scores predicted using the DNN might be strongly influenced by the age of the subject. Therefore, in order to evaluate the degree of influence of age on the DNN prediction, we compared the ground truth MMSE scores to the predicted MMSE scores using a second DNN, trained without the age parameter (Figure 3B). While the correlation coefficient of the second DNN's output compared with the ground truth MMSE scores decreased slightly from 0.85 to 0.75 (as expected), the correlation was still statistically significant (*p* < 0.001).

### Prediction Accuracy of the Deep Neural Network Model in the Binary Classification of MMSE

We then validated the DNN model as a binary classifier to detect the presence or absence of cognitive dysfunction using the MMSE threshold (a cut-off value of 23/24). The DNN model exhibited a high prediction accuracy for binary classification, with a sensitivity of 90% and specificity of 90%.

### Variable Importance Vis-à-vis the Prediction of MMSE Scores

We evaluated the variable importance vis-à-vis the prediction of MMSE scores. Table 5 shows the variable importance in DNN prediction. Interestingly, the variable with the highest importance “age,” also showed the highest correlation coefficient in Spearman's rank-order correlation. In addition, albumin, Hb, and RBC exhibited high importance in DNN prediction.

**Table 5:** Variable importance in deep learning prediction.

**Rank**	**Variable**	**Relative importance**
1	Age	1
2	Alb	0.59
3	PLT	0.55
4	Ht	0.54
5	Hb	0.53
6	K	0.53
7	BUN	0.49
8	RBC	0.48
9	MCV	0.45
10	UA	0.44

*MCV, mean corpuscular volume; MCH, mean corpuscular hemoglobin; MCHC, mean corpuscular hemoglobin concentration; BUN, blood urea nitrogen*.

### Validation of Prediction Accuracy of DNN Model

**Figure 4:**
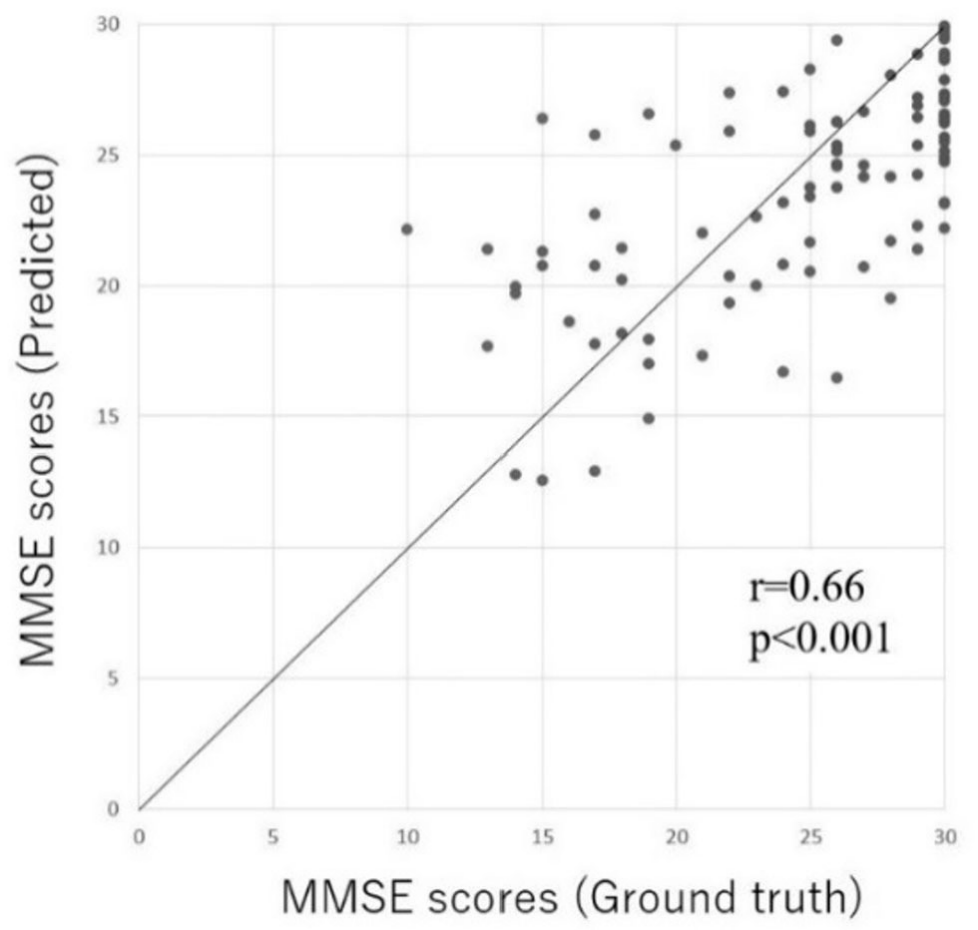
Scatter plot of the predicted MMSE scores and ground truth MMSE scores in the Inpatient and Heathy groups. The horizontal axis represents the measured MMSE score, while the vertical axis indicates the MMSE score predicted by the DL-based screening test.

In order to validate the prediction accuracy of the DNN model, we compared the predicted MMSE scores and ground truth MMSE scores in the Patient and Heathy groups. Figure 4 shows the scatter plots of the ground truth MMSE scores and those predicted by the DNN model. The predicted MMSE scores exhibited a significant positive correlation with the ground truth MMSE scores (*r* = 0.66, *p* < 0.001). The MAE was 3.43 with a standard deviation of 2.59.

**Figure 5:**
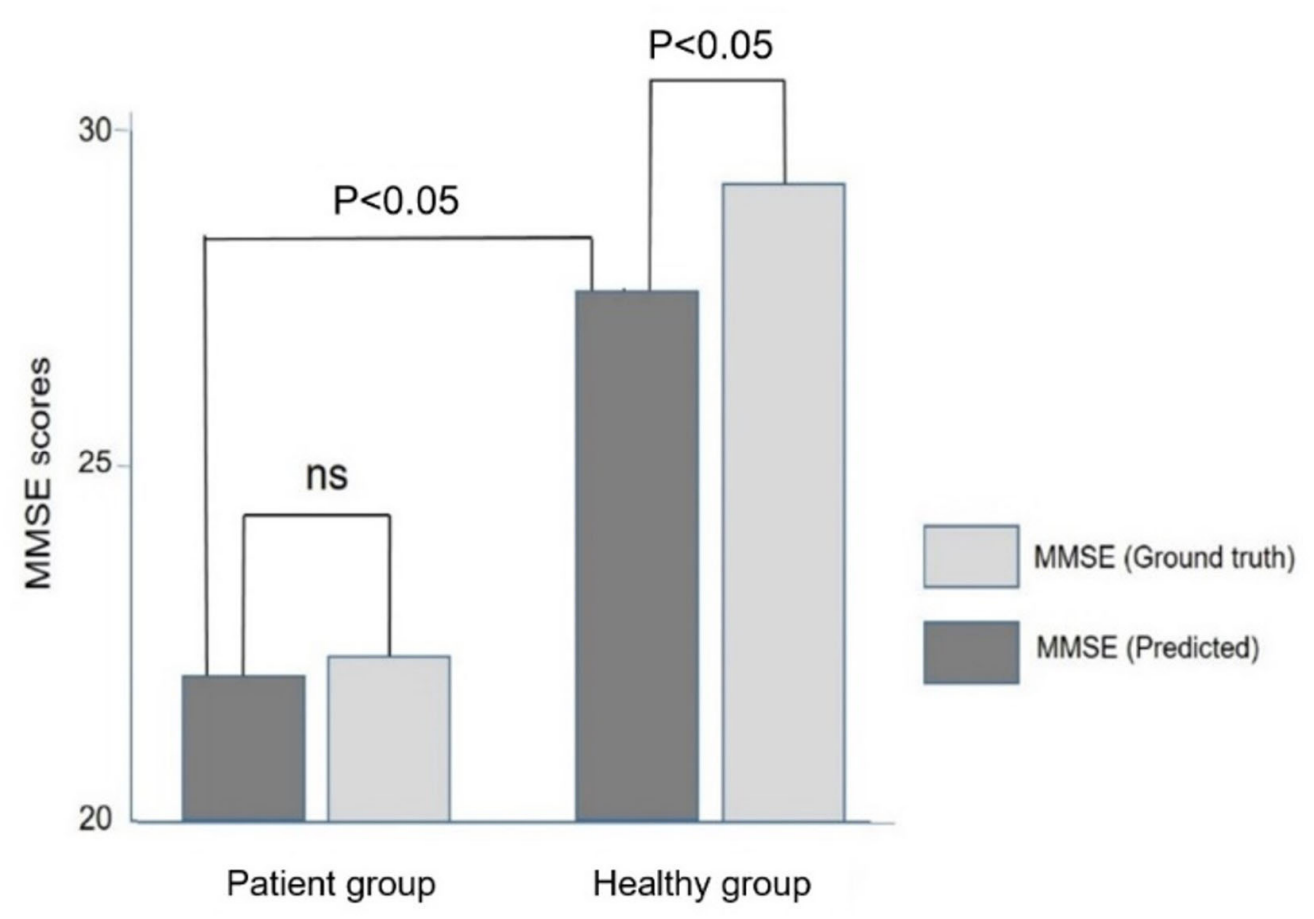
Comparison of the ground truth and predicted MMSE scores in the Patient and Healthy groups. Note that the predicted MMSE scores were lower than the ground truth MMSE scores in the healthy group.

Figure 5 compares the ground truth and predicted MMSE scores in the Inpatient and Heathy Groups. The ground truth MMSE scores (22.4 ± 5.4) in the Patient group were significantly lower than those in the healthy group (29.4 ± 1.3) (*p* < 0.05). The predicted MMSE scores (22.0 ± 3.9) in the Patient group were significantly lower than those in the Healthy group (27.6 ± 2.2) (*p* < 0.05), which was consistent with the relationship observed in the ground truth MMSE scores. There was no significant difference between the predicted (22.4 ± 5.4) and ground truth MMSE scores (22.4 ± 5.4) in the Patient group (*p* > 0.05). However, in the healthy group, the predicted MMSE scores (27.6 ± 2.2) were significantly lower than the ground truth scores (29.4 ± 1.3) (*p* < 0.05).

We evaluated the prediction accuracy of the DNN model in the two-class classification of MMSE (normal, MMSE scores ≥ 24; cognitive impairment, MMSE scores, ≤ 23). We observed a high prediction accuracy with a sensitivity of 75% and specificity of 87%.

### Clinical Experience Using the DNN Model

**Figure 6:**
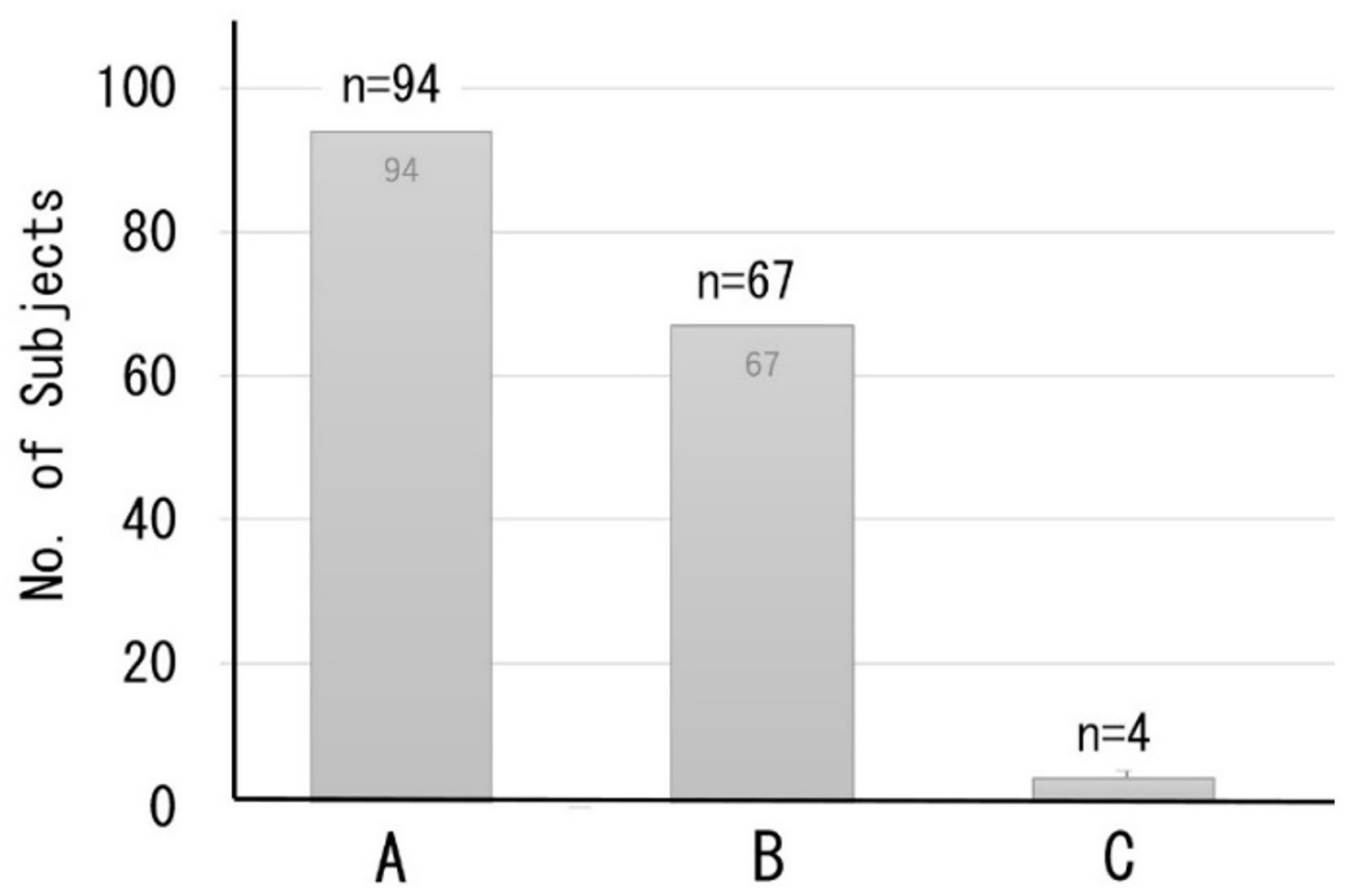
Distribution of the classification of cognitive function based on predicted MMSE scores. Class A (MMSE scores 27–30) = normal, B (MMSE scores 24–26) = suspected MCI, C (MMSE scores ≤23) = suspected dementia.

A total of 165 subjects chose the optional DNN-based dementia risk test at the time of their health examination; most subjects had a normal working life and attended the clinic as a regular health check imposed by the employer on the worker. Figure 6 shows the distribution of the classification of cognitive function based on the MMSE predicted by the DNN model. A total of 94 subjects were classified as Class A (normal), while 67 subjects were classified as Class B (suspected MCI). Only four cases were classified as Class C (suspected dementia).

**Figure 7:**
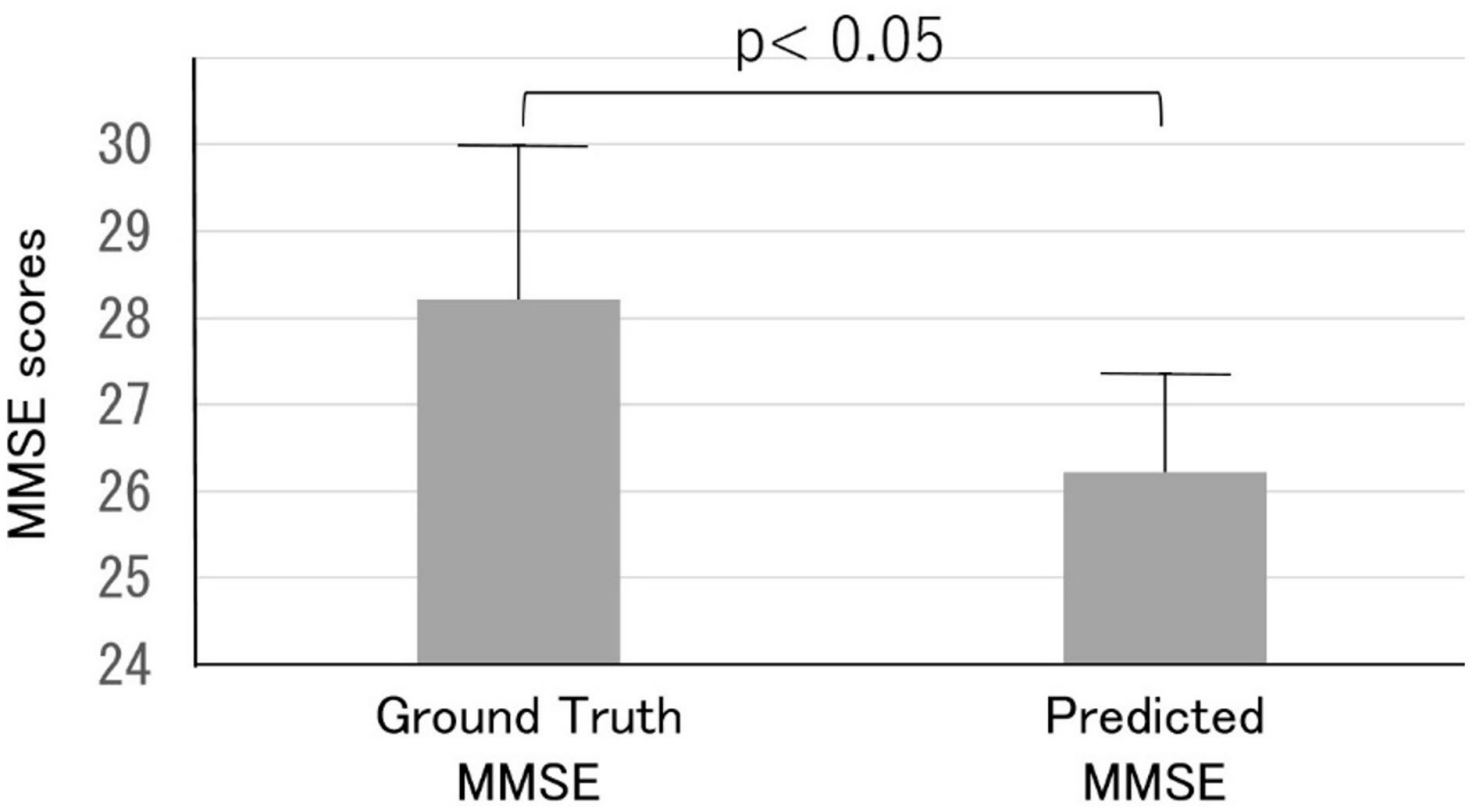
Comparison of the ground truth and predicted MMSE scores in the Health examination group. Note that the predicted MMSE scores were lower than the ground truth MMSE scores.

Thirty out of the 165 subjects (mean age, 56.3 ± 9.4 years) were readmitted to the dementia prevention outpatient clinic at the clinic and underwent a detailed examination of cognitive dysfunction involving various neuropsychological tests, including the MMSE, and imaging-based diagnosis using MRI. Most readmitted patients were classified as class B (suspected MCI) or lower. The predicted MMSE scores (26.2 ± 1.1) were significantly lower than the ground truth MMSE scores (28.2 ± 1.8, *p* < 0 0.05) (Figure 7).

However, in four cases, the predicted MMSE scores (26.9 ± 1.1) exceeded the ground truth MMSE scores (25.3 ± 0.5); the average difference in the MMSE score was −1.7 ± 0.8. Two of the subjects had a neurological disease, such as subarachnoid hemorrhage (SAH). One patient (male, 66 years old) had SAH due to cerebral aneurysm rupture 2 years before the DNN-based dementia risk test; MRA showed an aneurysm of the anterior communicating artery. The patient had undergone endovascular coiling. At the time of examination, there was no motor paralysis, but mild cognitive decline was observed (MMSE score = 25). The blood test data were within the normal range, and the predicted MMSE score was 27.5.

### Relationship Between Blood Data and MRI

Table 6 shows the correlations between blood data and the variables on VSRAD, which is an MRI-based brain atrophy index. We found that total protein, A/G ratio, and Cl significantly correlated with the variables on VSRAD.

**Table 6:** Correlation between blood data and MRI findings.

**Blood data**	**VSRAD**			
	**Severity**	**Brain Extent (%)**	**Extent**	**Ratio**
			**(%)**	
Total protein	*r* = 0.423^**^	ns	*r* = 0.412^*^	*r* = 0.368^*^
A/G ratio	*r* = 0.453^*^	*r* = 0.564^**^	*r* = 0.456^*^	*r* = 0.415^*^
CL	−0.43^*^	ns	*r* = −0.425^*^	*r* = −0.467^*^

* *p < 0.05*,

** *p < 0.01*.

## Discussion

### Prediction Accuracy of DNN Model

Originally inspired by biological neural networks, a DNN is capable of learning functional approximations to a wide variety of problems, including regression and classification. In many cases, a DNN model can be trained to predict with extremely high accuracy (16). Indeed, the present study demonstrated that the DNN-based screening method was able to predict the cognitive function expressed by MMSE scores with high accuracy based on basic blood test data for health examination. At first, we evaluated the prediction accuracy by LOOCV and found a significant correlation between the ground truth and predicted MMSE scores. It should be noted that the accuracy of the MMSE scores predicted by the DNN model was higher than that predicted by the logistic regression analysis in the two-class classification of normal and cognitive impairment (Tables 5, 6). In addition, the predictive accuracy of the DNN model was validated in subjects who were not included in the training of the DNN model. There was a significant positive correlation between the ground truth and predicted MMSE scores (*r* = 0.66, *p* < 0.001). Moreover, the binary classification based on MMSE scores showed a high prediction accuracy with a sensitivity of 75% and specificity of 87%. These results suggest that the DNN model allows us to predict cognitive dysfunction expressed by MMSE in elderly individuals with high accuracy based on basic blood test data, which does not include AD-related biomarkers such as amyloid β. Although the prediction accuracies were slightly lower than those in the LOOCV with the training group data, we believe that the DNN model can predict MMSE scores with high accuracy and can be applied in clinical screening tests of cognitive impairment.

### Mechanisms of Prediction of Cognitive Impairment Based on Basic Blood Test

It is not yet clear why the DNN model could predict cognitive function based on basic blood test data; however, the following two pathophysiological mechanisms should be considered. First, the vascular factor (i.e., vascular cognitive impairment, VCI) plays an important role in the underlying mechanism of the cognitive impairment in the subjects for training of the DNN model (7–9). The VCI is based on atherosclerosis which is caused by lifestyle diseases. Therefore, basic blood data may predict the degree of atherosclerosis based on the basic blood data which reflect lifestyle diseases. Another possible mechanism is systemic metabolic disorders which could affect cognitive function. Recent studies demonstrated that cognitive function could be affected by various systemic disorders, including malnutrition (11) and anemia (12). Indeed, we observed that albumin and A/G ratio exhibited significant positive correlations with the MMSE scores; albumin also showed a high importance in the prediction of MMSE scores by the DNN model. In addition, RBC and Hb showed significant positive correlations with the MMSE scores, and Ht and MCV showed high importance in the prediction by the DNN model. In addition, it has been reported that the following systemic disorders could affect cognitive function; liver dysfunction (27), abnormal lipid metabolism (13), renal dysfunction (15), abnormal purine metabolism (14), abnormal electrolytes (Na, Cl) (28), and platelets (29). These systemic disorders were consistent with the blood test items that correlated with MMSE scores and/or were the important variables in the prediction by the DNN model.

### Mechanisms of Cognitive Dysfunction in Elderly People

**Figure 8:**
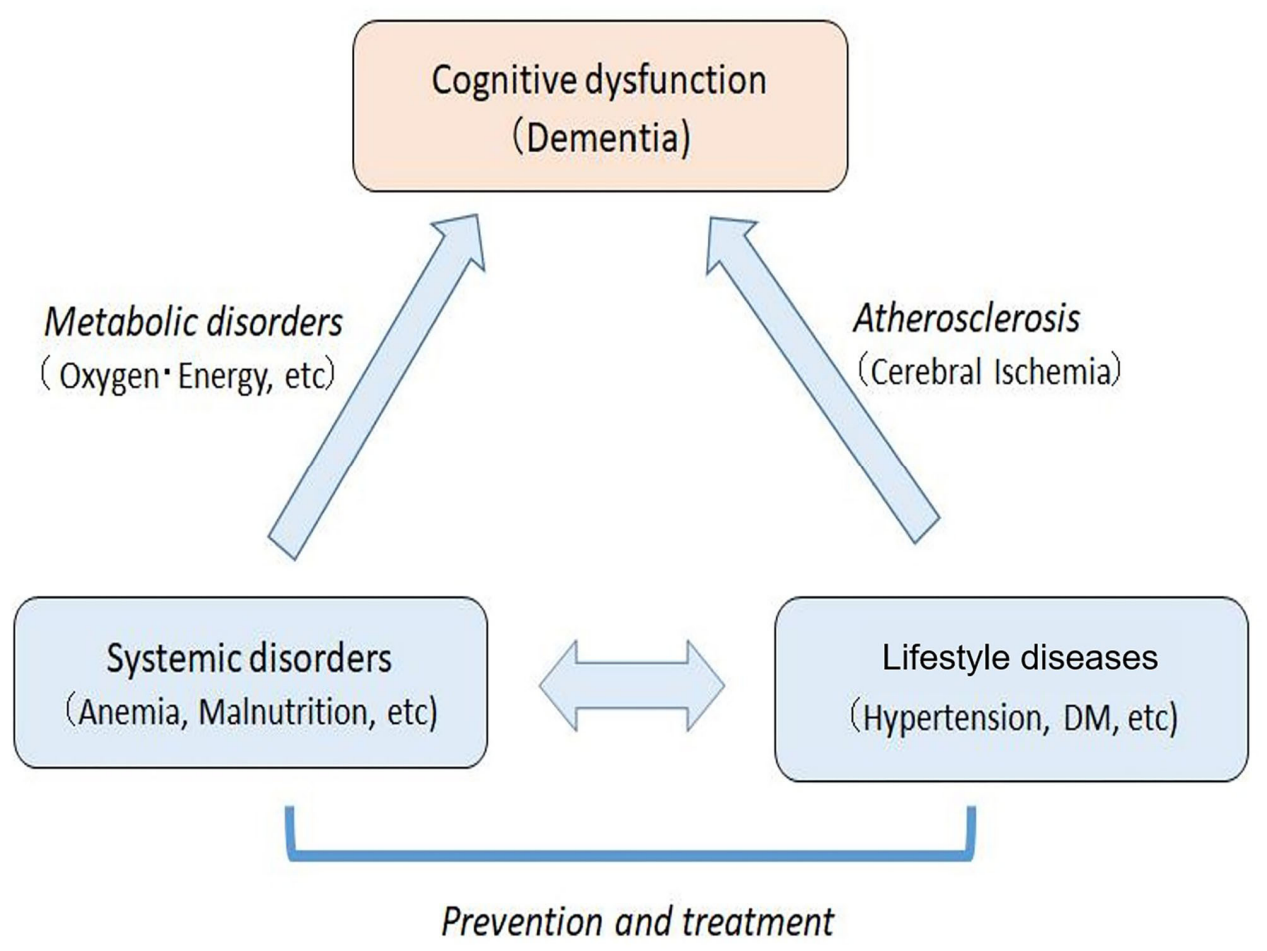
Mechanisms of cognitive dysfunction in the elderly with systemic metabolic disorders and arteriosclerosis based on lifestyle diseases. The systemic disorders can be prevented and treated, which leads to the prevention and improvement of cognitive impairment in elderly people.

Based on the reported studies and present study, we suggest that cognitive dysfunction in elderly people may be caused by a joint action of systemic metabolic disorders (e.g., energy and oxygen metabolism disorders) and cerebral circulation disorder due to arteriosclerosis based on lifestyle diseases (Figure 8). This paradigm suggests that the dementia of elderly individuals with lifestyle diseases and metabolic disorders could be regarded as a systemic disease rather than a brain disease localized to the central nervous system. Currently, it is difficult to treat the brain disorders of dementia. However, systemic metabolic disorders including lifestyle diseases can be treated, which leads to prevention or delay of the onset of cognitive disorders in the elderly. It should be emphasized that vascular factors are not limited to vascular dementia, but also play a role in the onset of AD (10). Therefore, treatments of vascular factors could reduce the onset of both vascular dementia and AD.

### Effects of Aging on Prediction by DNN Model

The predicted MMSE scores were lower than the ground truth MMSE scores in the Healthy group (*p* < 0.05), while there was no such difference in the Patient group. There were the following differences in the background of the subjects between the two groups. First, the average age of the Healthy group (62.0 ± 8.6 years) was significantly lower than that of the Patient group (73.6 ± 11.0 years, *p* < 0.05). Second, the subjects in the Healthy group less commonly had lifestyle diseases than those in the Inpatient group. Finally, the subjects in the Healthy group regularly exercised at the sports gym; similar behavior was not seen in the patient group. These differences suggest that subjects in the Healthy group might be less affected by atherosclerosis than those in the Patient group. Similar discrepancies between the predicted and ground truth MMSE scores were observed in the Health examination group. It should be noted that the subjects in the Health examination group were younger (56.3 ± 9.4 years) than those in the Inpatient group (73.6 ± 11.0 years), suggesting that a larger proportion of subjects in the Health examination group might not have atherosclerosis than that in the Patient group.

Based on these observations, we suggest that the difference between the predicted and ground truth MMSE scores was caused by the training group of the DNN model. The DNN model in the present study was trained in elderly patients with cerebrovascular diseases due to atherosclerosis. When the DNN model was applied to younger subjects with less advanced arteriosclerosis, the DNN model might predict the same MMSE score as the elderly who were used for training of the DNN model, if the blood data were the same, resulting in lower predicted MMSE scores than the ground truth in younger subjects. Therefore, applying the DNN model to younger subjects (e.g., 40–60 years old) may predict future cognitive impairment after the onset of atherosclerosis.

### Relationship Between Blood Data and MRI

Interestingly, there were significant correlations between the blood data (e.g., total protein, A/G ratio, and Cl) and the variables on VSRAD, which is an MRI index of brain atrophy (23, 24). Although the underlying mechanisms are not yet clear, these observations suggest that systemic metabolic disorders could affect the anatomical structure of the brain. In addition, the correlation between the blood data and VSRAD suggests that DNN may be able to predict changes in the anatomical structure of the brain detected by MRI based on basic blood test data. If MRI findings such as VSRAD can be predicted from the general blood test data, unnecessary MRI examinations will be less frequently performed, which will provide medical and economic advantages. Further studies are necessary to clarify whether deep learning can predict MRI findings based on basic blood test data.

### Advantages of DNN-Based Screening Test

When applying the DNN model to a screening test for cognitive impairment, the following advantages may be considered. First, one of the advantages is that only blood data values are used for the DNN-based screening test. Therefore, this test can be used as an inexpensive mass screening test for dementia. In addition, by entering blood data values into the smartphone, it is possible to use the smartphone for personal risk assessment of cognitive impairment. These advantages may contribute to the early diagnosis of MCI and dementia. Second, as discussed above, when this method is applied to middle-aged subjects, the result may be able to predict cognitive impairment when they become older in the future. Finally, this method may contribute not only to screening tests for dementia, but also to personalized care for the prevention of dementia. That is, the blood test data reflect systemic metabolic abnormalities in each individual; thus, it is possible to improve their lifestyle based on the abnormal blood data (11–15, 25–27). Personalized care not only increases the effectiveness of interventions but also enhances lifestyle incentives. Lifestyle improvement is not long-lasting for the purpose of improving lifestyle-related diseases, but long-lasting for the purpose of reducing the risk of dementia.

### Clinical Application of DNN Model to Medical Care of Dementia

**Figure 9:**
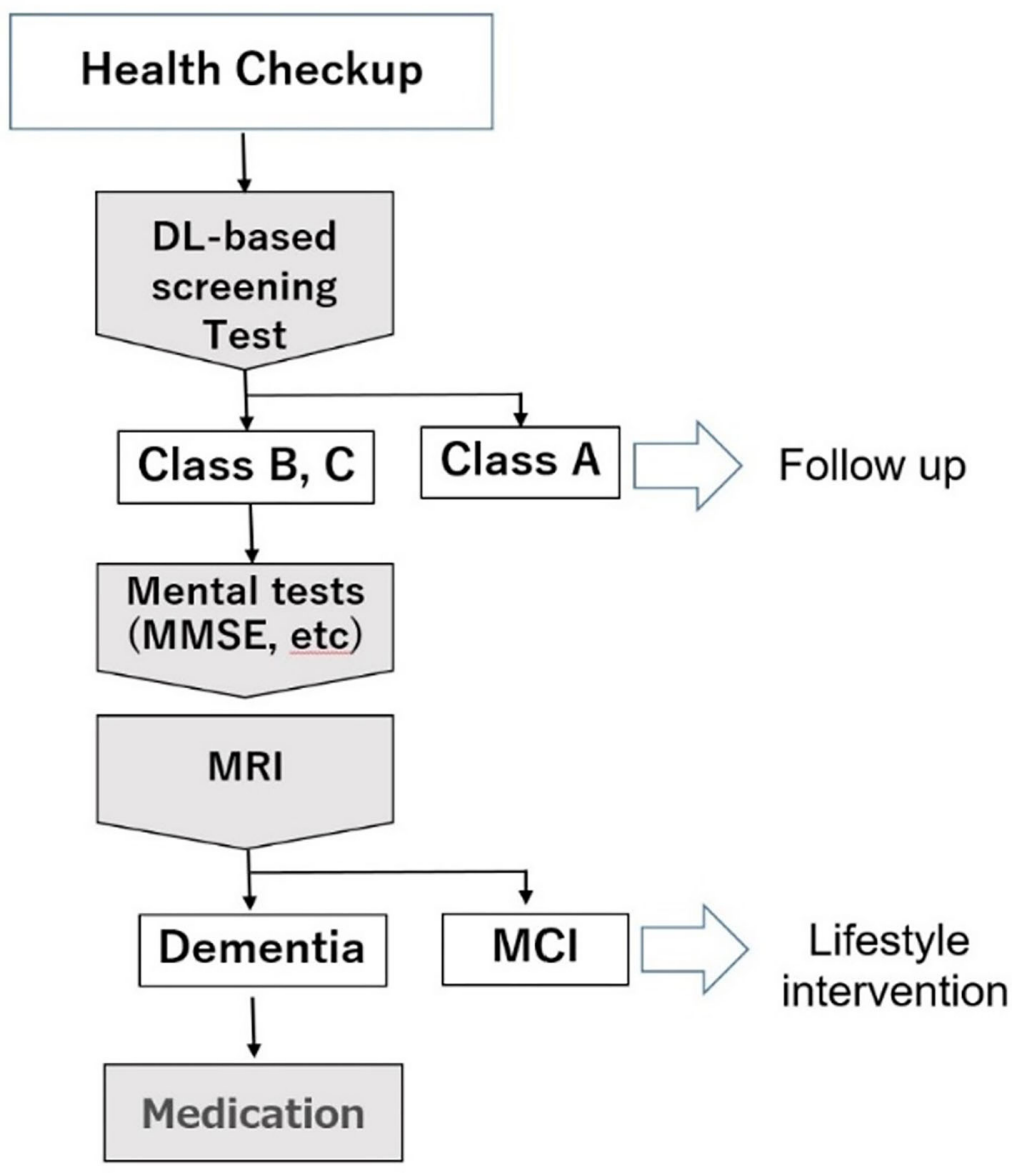
Medical examination procedure using the DNN-based screening test.

Figure 9 shows the consultation procedure at the Tokyo Clinic using the DNN-based screening test. First, the test is optionally performed in general health examination. Those classified as Class A will receive general life guidance, while those assessed as Class B (suspected MCI) or Class C (suspected dementia) will be advised to visit the outpatient clinic for further consultation. In the outpatient clinic, the MMSE and other neuropsychological tests will be performed on these patients. If their cognitive function is not impaired or MCI, their systemic metabolic disorders that are risk factors for dementia will be treated by a general practitioner. If there is an apparent cognitive impairment, MRI, and other imaging modalities will be performed. Patients diagnosed with dementia will be treated by a dementia specialist.

### Limitations of DNN-Based Screening Test

Finally, the limitations of the present study should be discussed. First, most of the patients for training of the DNN model had received medical treatments for their lifestyle diseases and cerebrovascular diseases at the time of this study. Therefore, the blood data tended to exhibit normal values or only small abnormalities, resulting in poor correlations with MMSE scores and less variable importance in the DNN model. Second, the DNN model may miss cognitive impairment in subjects whose cause is limited to the brain. Therefore, patients with the juvenile form of Alzheimer's disease who have no systemic disorders may be overlooked by this method. The use of functional brain monitoring such as near-infrared spectroscopy (NIRS) may be useful to avoid such misdiagnosis (29–31). Further evaluation is needed to validate the DNN model for clinical application. In particular, the false-positive/false-negative ratio should be assessed more accurately using a large number of subjects. Finally, the input layer of the DNN model primarily uses only blood data and subject's age, but does not include physical findings (body mass index, blood pressure, etc.) or medical history. The prediction accuracy may be improved by increasing the input layer items. In order to establish the present method as a mass screening test for dementia, further studies are needed to resolve these limitations.

### Summary

We have developed a DNN model which allows to predict cognitive impairment expressed by MMSE scores. This method is based on the idea that cognitive impairment in the elderly is caused by systemic metabolic disorders such as lifestyle diseases, and thus, uses basic blood test data of health examinations that do not include biomarkers for dementia such as amyloid β. Therefore, it can be applied to mass screening tests for dementia and can be used to test for dementia with a smartphone. This method may contribute not only to early detection of cognitive decline such as MCI, but also to personalized care for the prevention of dementia based on the abnormal blood data that reflect individual risk factors for cognitive impairment. Finally, it should be emphasized that the combination of the DNN-based screening test and behavioral changes may contribute to the prevention of dementia and health economics in the elderly.

## Data Availability Statement

The raw data supporting the conclusions of this article will be made available by the authors, without undue reservation.

## Ethics Statement

The studies involving human participants were reviewed and approved by The Life Science Research Ethics and Safety of the University of Tokyo (Approval Number: 19-318). The patients/participants provided their written informed consent to participate in this study.

## Author Contributions

KS designed the study and wrote the initial draft of the manuscript. LH and KO contributed to the analysis and interpretation of data and assisted in the preparation of the manuscript. All authors approved the final version of the manuscript and agreed to be accountable for all aspects of the work by ensuring that questions related to the accuracy and integrity of any part of the work are appropriately investigated and resolved.

## Conflict of Interest

The authors declare that the research was conducted in the absence of any commercial or financial relationships that could be construed as a potential conflict of interest.
